# Anti-thymocyte globulin (ATG)- or alemtuzumab-based graft-versus-host disease prophylaxis in reduced-intensity conditioning allogeneic hematopoietic cell transplantation (HCT) for patients 40 years and older with acute lymphoblastic leukemia in first complete remission: a study from the EBMT Acute Leukemia Working Party

**DOI:** 10.1038/s41409-026-02805-4

**Published:** 2026-03-06

**Authors:** Gesine Bug, Myriam Labopin, Jennifer L. Byrne, Stephan Mielke, Kim Orchard, Shankara Paneesha, Victoria Potter, Didier Blaise, Caroline Besley, John A. Snowden, Ibrahim Yakoub-Agha, Andrew Clark, Charles Crawley, Alexandros Spyridonidis, Jaime Sanz, Eolia Brissot, Sebastian Giebel, Fabio Ciceri, Mohamad Mohty

**Affiliations:** 1 Goethe University, Department of Medicine 2, Frankfurt am Main, Germany; 2 Sorbonne University, Department of Hematology, Saint Antoine Hospital, INSERM, Paris, France; 3 Nottingham University Hospitals Trust, Nottingham, UK; 4 Department of Cellular Therapies and Allogeneic Stem Cell Transplantation (CAST), Karolinska CCC and ATMP Center, Stockholm, Sweden; 5 Wessex Blood and Marrow Transplantation and Cellular Therapy, Dept of Haematology, University Hospital Southampton NHSFT, Southampton, UK; 6 University Hospitals Birmingham NHS Foundation Trust, Queen Elizabeth Medical Centre, Edgbaston, Dept. of Haematology, Birmingham, UK; 7 Kings College Hospital, Dept. of Haematological Medicine, King’s Denmark Hill Campus, London, UK; 8 Transplantation and Cellular Immunotherapy, Department of Hematology, Institut Paoli Calmettes, Management Sport Cancer Lab, Aix Marseille University, Marseille, France; 9 University Hospitals Bristol and Weston NHSFT, Bristol, UK; 10 Department of Haematology, Sheffield Teaching Hospitals NHS Trust, Sheffield, UK; 11 Division of Clinical Medicine, School of Medicine and Population Health, The University of Sheffield, Sheffield, UK; 12 CHU de Lille, Univ de Lille, INSERM, Lille, France; 13 Bone Marrow Transplant Unit, Beatson, West of Scotland Cancer Centre, Glasgow, UK; 14 Department of Haematology, Addenbrookes Hospital, Cambridge, UK; 15 Bone Marrow Transplantation Unit and Institute of Cellular Therapy, University of Patras, Patras, Greece; 16 Hematology, University Hospital La Fe, Madrid, Spain; 17 Maria Sklodowska-Curie National Research Institute of Oncology, Gliwice Branch, Gliwice, Poland; 18 Hematology and Bone Marrow Transplant Unit, IRCCS San Raffaele Scientific Institute, Milan, Italy

**Keywords:** Acute lymphocytic leukaemia, Bone marrow transplantation

## Abstract

For patients with high-risk acute lymphoblastic leukemia (ALL), allogeneic hematopoietic cell transplantation (HCT) remains standard of care. In the setting of an HLA-matched unrelated donor HCT, in vivo T-cell depletion (TCD) for prophylaxis of graft versus host disease (GVHD) relies on anti-thymocyte globulin (ATG) in Europe and alemtuzumab in the UK. In a retrospective study from the EBMT registry, we pair-matched 90 ALL patients aged ≥40 years transplanted in CR1 according to age (median 56 years) and ALL subtype (37.8% Ph-negative B-ALL, 46.7% Ph-positive B-ALL, 15.6% T-ALL). Reduced-intensity conditioning included fludarabine/melphalan (94.4%) in the alemtuzumab and fludarabine/busulfan (36.7%), fludarabine/total body irradiation (21.1%), fludarabine/melphalan (14.4%) and thiotepa/busulfan/fludarabine (13.3%) in the ATG group. Two-year leukemia-free and overall survival were similar between groups (Alemtuzumab: 56.4% vs ATG: 50.7%, HR 0.82, *p* = 0.34, and 62.7% vs 62.9%, HR 0.91, *p *= 0.67), as were cumulative incidence of relapse (23.7% vs 23.9%, HR 0.89, *p* = 0.69) and non-relapse mortality (19.9% vs 25.4%, HR 0.75, *p* = 0.32), resulting in similar GVHD- and relapse-free survival (GRFS) of 48.9% vs 42.1%, HR 0.8, *p* = 0.24. With GVHD and infections as main reasons for death in both groups, we conclude that both IS strategies are both safe for RIC HCT of these ALL patients.

## Introduction

For adult patients with high-risk acute lymphoblastic leukemia (ALL), allogeneic hematopoietic cell transplantation (HCT) in first complete remission remains standard of care resulting in 5-year overall survival rates of 50–70% in younger patients [1–4],. To address age-dependent increases in toxicity and non-relapse mortality (NRM) [5], reduced intensity conditioning (RIC) has been explored in patients above the age of 40 or 45 years [6, 7],. In these retrospective studies, NRM of around 20% was counterbalanced by a high relapse incidence of >40% and an unsatisfactory 5-year OS of 40%.

Recently, the largest prospective study of RIC HCT in ALL to date, a single-arm sub-study embedded in the UKALL14 randomized trial, has demonstrated favorable 4-year OS of 55% in the 249 ALL patients aged 41–65 years who underwent a matched sibling or unrelated donor HCT with fludarabine and melphalan as RIC [8]. In this uniformly treated cohort of patients, alemtuzumab, a monoclonal antibody directed against CD52, was used for in vivo T cell depletion (TCD). As CD52 is expressed on B- and NK cells, alemtuzumab also has a potent in-vivo depleting effect on these immune effector lineages [9]. This FMA transplant regimen has been pioneered to combine potent antileukemic activity with efficacious GVHD prophylaxis in older or comorbid patients with high-risk myeloid diseases [10, 11].

Outside of the UK, in vivo TCD is mostly based on rabbit anti-thymocyte globulin (ATG), a polyclonal immunoglobulin, obtained by immunization of rabbits with human thymocytes or Jurkat T cell line [12]. The efficacy of preventing severe acute and extensive GVHD following mostly myeloablative conditioning and matched unrelated donor HCT in patients with various hematologic diseases has been demonstrated in several randomized clinical trials, with heterogeneous impact on OS [13–15]. In retrospective studies focusing on ALL, use of ATG has been associated with reduced chronic GVHD without impacting overall survival [16, 17].

To date, the very few studies comparing ATG and alemtuzumab in hematologic malignancies have included no or very few patients with ALL [18–22]. The paucity of data on the relative contributions of an ATG-based RIC regimen versus FMA conditioning to incidence and severity of GVHD, risk of relapse, NRM and survival in patients with high-risk ALL in CR1 prompted us to retrospectively compare these two strategies using the EBMT registry data.

## Methods

### Data collection

Data for this retrospective multicenter study were retrieved from the registry of the Acute Leukemia Working Party (ALWP) of the European Society for Blood and Marrow Transplantation (EBMT), a nonprofit, scientific society representing >600 transplant centers, mainly located in Europe. Centers commit to reporting all consecutive HCTs and follow-ups once a year. Data are entered, managed, and maintained in a central database and validated by verification of the computer printout of the entered data, cross-checking with the national registries, and on-site visits to selected teams. All patients gave informed consent authorizing the use of their personal information for research purposes. This study was approved by the ALWP of the EBMT institutional review board and conducted per the Declaration of Helsinki and Good Clinical Practice guidelines.

### Criteria for patient selection

Patient selection was based on the following criteria: (1) Patients with ALL in CR1 (2) aged 40 years or above who had received (3) a first allogeneic HCT following reduced-intensity or toxicity-reduced conditioning between January 2010 - December 2021 with  (4) PBSC from a matched unrelated donor [4]. T-cell depletion as part of the immunosuppressive regimen was based on ATG (Grafalon®, Neovii Pharmaceuticals (formerly commercialized as ATG Fresenius) or Thymoglobulin®, Sanofi) or alemtuzumab (Campath®, Genzyme) [5]. Administration of ex vivo TCD or post-transplantation cyclophosphamide was not permitted.

### Statistical analysis

The study objective was to compare ATG- and alemtuzumab-based GVHD prophylaxis in RIC HCT for ALL in CR1 in relation to survival outcomes and the efficacy of preventing GvHD. Primary endpoint was overall survival, secondary endpoints were cumulative incidence (CI) of relapse and leukemia-free survival (LFS), CI of acute and chronic GvHD and grade III-IV acute GVHD/ severe chronic GVHD, relapse-free survival (GRFS). Acute and chronic GVHD were diagnosed according to the modified Glucksberg criteria and modified Seattle criteria, respectively. Probabilities for OS, LFS and GRFS were calculated using Kaplan–Meier estimates, and cumulative incidence (CI) curves for relapse, NRM, acute and chronic GVHD using a competing risk model: relapse and death are competing together, i.e. relapse is the competing event for NRM and death without relapse the competing event for relapse, whereas relapse and death were competing risks for GVHD. Univariate analyses were performed using the log-rank test for LFS, OS, and GRFS, and Gray’s test for CI estimates. Patient, disease, and transplant characteristics were compared by using the χ^2^ or Fisher’s exact test for categorical variables and the Mann–Whitney or Kruskal–Wallis test for continuous variables [23, 24]. For propensity score matching, exact matching was performed in a 1:1 ratio for the subtype of ALL, while the nearest neighbor was chosen for age at transplant. All tests were two-sided with the type 1 error rate fixed at 0.05. SPSS 27.0 (IBM Corp., Armonk, NY, USA) and R 4.0.2 (R Core Team 2020. R: a language and environment for statistical computing. R Foundation for Statistical Computing, Vienna, Austria, https://www.Rproject.org/)), were used for all statistical analyses.

## Results

### Patients and transplant procedures

We analyzed 357 patients with ALL of whom 236 received ATG and 121 alemtuzumab for TCD. Comparison of demographics and transplant modalities revealed an imbalance between ATG- and alemtuzumab-treated patients regarding age, performance status, proportion of BCR::ABL1 positive patients, CMV status and the ratio of female donors to male recipients (Supplementary Table 1). Briefly, patients in the ATG group were significantly older (median age 60.4 [range, 40–72] vs 53.6 [range, 40–71] years, *p* < 0.0001). The underlying diagnosis was Philadelphia (Ph)-negative B ALL (23.7% with ATG and 47.1% with alemtuzumab), Ph-positive ALL (60.0% and 34.7%, respectively) and T ALL (15.7% and 18.2%, respectively), *p* < 0.0001. To address this imbalance, we conducted propensity score matching with exact matching for subtype of ALL and nearest neighbor matching for age at HCT.

### Pair-match analysis on propensity score

We pair-matched 90 patients in each group with Ph-negative B ALL (37.8%), Ph-positive B ALL (46.7%,) and T ALL (15.6%) and a median age of 56 [range, 40–70] years in the ATG and 55.7 [range, 41–71] years in the alemtuzumab group (Table 1). Median year of HCT was 2016 and time from diagnosis to transplant was 6 months in both groups.

**Table 1:** Patient and transplant characteristics of the pair-match population.

		ATG (*n* = 90)	Alemtuzumab (*n* = 90)	*P*
Median follow-up (months)	Median [IQR]	36.65 [26.69–53.34]	49.24 [46.44–58.27]	0.50
Patient age (years)	Median (min-max) [IQR]	56 (40.6–70.3) [49.7–60.2]	55.7 (41.1–71) [49.8–60.1]	0.76
Diagnosis	Ph negative B ALL	34 (37.8%)	34 (37.8%)	1
	Ph positive B ALL	42 (46.7%)	42 (46.7%)	
	T ALL	14 (15.6%)	14 (15.6%)	
Year transplant	Median (min-max)	2016 (2010–2021)	2016 (2010–2021)	0.36
Time diagnosis to HCT (mo)	Median (min-max) [IQR]	6.3 (2.7–20.2) [5.4–8]	6 (3.6–21.4) [4.9–7.6]	0.41
Patient sex	Male	40 (44.4%)	42 (46.7%)	0.76
	Female	50 (55.6%)	48 (53.3%)	
Donor sex	Male	73 (81.1%)	65 (74.7%)	0.3
	Female	17 (18.9%)	22 (25.3%)	
	Missing	0	3	
Female to male combination	no F- > M	85 (94.4%)	76 (85.4%)	0.044
	F- > M	5 (5.6%)	13 (14.6%)	
	Missing	0	1	
Karnofsky score	<90	25 (29.8%)	32 (37.6%)	0.28
	≥90	59 (70.2%)	53 (62.4%)	
	Missing	6	5	
MRD pre HCT	MRD negative	39 (69.6%)	24 (64.9%)	0.63
	MRD positive	17 (30.4%)	13 (35.1%)	
	Missing	34	53	
GVHD prophylaxis	CSA	10 (11.1%)	67 (74.4%)	<0.0001
	Tacrolimus	1 (1.1%)	6 (6.7%)	
	CSA + MTX	31 (34.4%)	11 (12.2%)	
	Tacrolimus+MTX	1 (1.1%)	0	
	CSA + MMF	34 (37.8%)	5 (5.6%)	
	CSA + MTX + MMF	2 (2.2%)	0	
	Tacrolimus + MTX + MMF	7 (7.8%)	0	
	Tacrolimus + Sirolimus	2 (2.2%)	0	
	Other	0	1 (1.1%)	
Patient CMV	Negative	23 (25.6%)	44 (50%)	0.0008
	Positive	67 (74.4%)	44 (50%)	
	Missing	0	2	
Donor CMV	Negative	59 (67.8%)	49 (55.7%)	0.099
	Positive	28 (32.2%)	39 (44.3%)	
	Missing	3	2	
Conditioning regimen	BuCy	1 (1.1%)	0	<0.0001
	BuFlu	33 (36.7%)	0	
	TBF	12 (13.3%)	0	
	FluMel	13 (14.4%)	85 (94.4%)	
	FTM	1 (1.1%)	0	
	FluTreo	3 (3.3%)	0	
	FluCy	2 (2.2%)	0	
	Cy-TBI	0	1 (1.1%)	
	Flu-TBI	19 (21.1%)	0	
	Thiotepa-based	1 (1.1%)	2 (2.2%)	
	Clofarabine-based	2 (2.2%)	0	
	Other chemotherapy	3 (3.3%)	2 (2.2%)	

*ATG* anti-thymocyte globulin, *HCT* hematopoietic cell transplantation, *ALL* acute lymphoblastic leukemia, *Ph* Philadelphia, *MRD* measurable residual disease, *CSA* cyclosporine A, *MTX* methotrexate, *MMF* mycophenolate mofetil, *Bu* busulfan, *Cy* cyclophosphamide, *Flu* fludarabine, *TBF* thiotepa, busulfan, fludarabine, *Mel* melphalan, *Treo* treosulfan, *TBI* total body irradiation.

Patients in the alemtuzumab group almost exclusively received fludarabine/melphalan (Flu/Mel) conditioning (94.4%), whereas conditioning regimens in the ATG group were more heterogeneous and included fludarabine/busulfan (36.7%), fludarabine/total body irradiation (TBI, 21.1%), Flu/Mel (14.4%) and thiotepa/busulfan/fludarabine (13.3%).

GVHD prophylaxis was based primarily on cyclosporine A, either as single agent (74.4% and 11.1% in alemtuzumab and ATG groups, respectively) or combined with methotrexate (12.2% and 34.4%) or mycophenolate mofetil (MMF, 5.6% and 37.8%). Tacrolimus +/− methotrexate or MMF was used in 6.7% and 10.0% of patients, respectively. Doses of alemtuzumab and ATG administered are given in Table 2.

**Table 2:** Breakdown of ATG and alemtuzumab doses administered.

Dose (mg/Kg BW)	ATG *N* (%)	Dose (mg)	Alemtuzumab *N* (%)
Thymoglobulin 2.5–<4.0	7 (7.8)	<50	9 (10)
4.0–<7.5	42 (46.7)	50–60	77 (85.6)
7.5–<15	11 (12.2)	>60	2 (2.2)
Grafalon 16–<25	3 (3.3)	missing	2 (2.2)
25–<45	13 (14.4)		
45–<60	3 (3.3)		
missing	11 (12.2)		

The brand of ATG was not captured in the database. However, dosing in excess of 15 mg/kg body weight is not applicable to Thymoglobulin, and these patients were considered to have received Grafalon [23].

### Outcomes

**Fig. 1:**
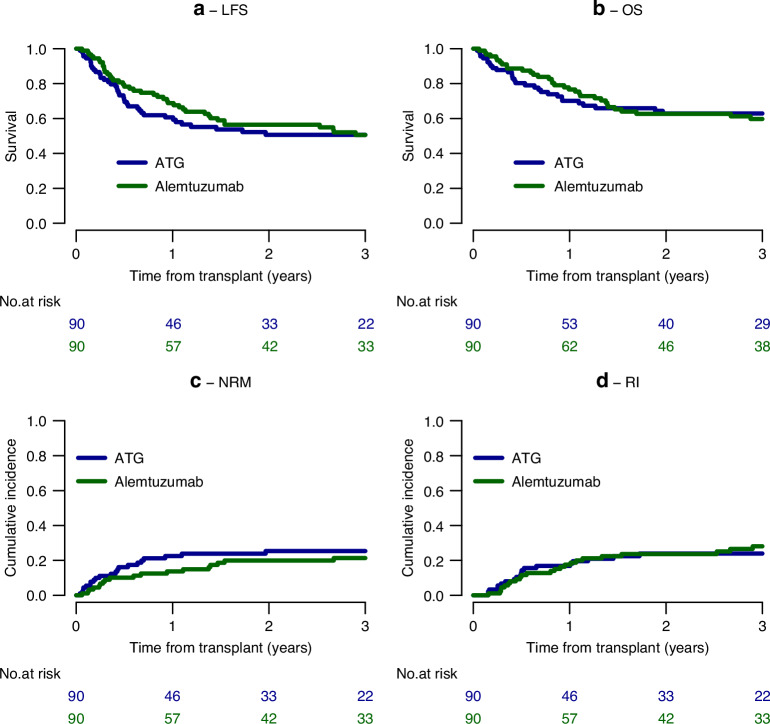
Outcome of patients according to in vivo T cell depletion. **a** Leukemia-free Survival; **b** Overall Survival; **c** Non-relapse Mortality; **d** Relapse Incidence.

All but four patients engrafted (alemtuzumab: *n* = 3; ATG: *n* = 1). No difference between groups was observed in terms of leukemia-free survival (LFS) and overall survival (OS) at two years (Alemtuzumab: 56.4% vs ATG: 50.7%, HR 0.82, *p* = 0.34, and 62.7% vs 62.9%, HR 0.91, *p* = 0.67, respectively, Fig. 1a, b). Non-relapse mortality (NRM, 19.9% vs 25.4%, HR 0.75, *p* = 0.32, Fig. 1c) and cumulative incidence (CI) of relapse (23.7% vs 23.9%, HR 0.89, *p* = 0.69, Fig. 1d) resulted in similar GVHD- and relapse-free survival (GRFS) of 48.9% vs 42.1%, HR 0.8, *p* = 0.24 in alemtuzumab and ATG-treated patients, respectively (data not shown). Leukemia recurrence (42.4% vs 25%), GVHD (27.3% vs 12.4%) and infections (12.1% vs 21.9%) were reported as the most frequent reasons for death in both the alemtuzumab and ATG group, respectively. Alemtuzumab and ATG-based TCD were associated with an equally low CI of severe acute GVHD (8.0% vs 6.8%, HR 1.11, *p* = 0.84, respectively) and extensive chronic GVHD (8.4% vs 12.5%, HR 0.5, *p* = 0.15, respectively) (Table 3).

**Table 3:** Cumulative incidence of acute or chronic GVHD.

	180 days		2 years	
	Acute GVHD II-IV	Acute GVHD III-IV	Chronic GVHD	Extensive chronic GVHD
ATG	21.6% [13.7–30.7]	6.8% [2.8–13.4]	29.1% [19–40]	12.5% [6–21.3]
Alemtuzumab	23.9% [15.6–33.3]	8% [3.5–14.8]	28.8% [19.1–39.3]	8.4% [3.4–16.3]
HR (95% CI) Alemtuzumab vs ATG	0.99 (0.56–1.77)	1.11 (0.4–3.1)	0.83 (0.45–1.52)	0.5 (0.19–1.28)
*P* value (cluster = pairs)	0.98	0.84	0.54	0.15

*ATG* anti-thymocyte globulin, *HR* hazard ratio, *CI* confidence interval, *GVHD* graft-versus-host disease.

## Discussion

ATG and alemtuzumab are mechanistically similar types of in vivo TCD, which have not been directly compared in the setting of RIC transplantation from a MUD for patients with ALL. Using a pair-matched design, our study demonstrates that alemtuzumab and ATG-based TCD is associated with a similarly low incidence of severe acute GVHD and extensive chronic GVHD and shows no significant difference with respect to CIR, NRM, LFS and OS.

Since most alemtuzumab-treated patients in the registry (113 of 121) were enrolled in the UKALL14 sub-study [8], we aimed to provide a comparison with a contemporaneous group of patients receiving ATG for MUD HCT who met the key inclusion criteria of UKALL14 and restricted our study population to patients ≥40 years with ALL in CR1. Despite this selection, the ATG and alemtuzumab cohorts displayed considerable differences in patient characteristics and conditioning regimens. This prompted us to perform propensity score matching to compare two subgroups better balanced in key prognostic features including age, Karnofsky performance status, sex and ALL subtype. Median follow-up was sufficiently long to allow robust outcome assessment and was not significantly different between the ATG and alemtuzumab cohorts. Compared to the patients in the UKALL14 sub-study, our alemtuzumab cohort included a higher proportion of MUD transplants (100% vs. 62%) and of BCR::ABL1 positive patients (47% vs. 25%) and had a higher median age (55.7 years vs. 50 years). Despite these differences, 2-year OS, CIR and CI of NRM in the overall study population were consistent with the survival outcome reported by Marks et al. [8]. While the overall incidence of acute and chronic GVHD did not differ significantly between the two cohorts in our study, our alemtuzumab cohort experienced a higher rate of acute GVHD II-IV (23.9% vs 12%) but lower incidence of overall (28.8% vs 37%) and extensive chronic GVHD (8.4% vs. 22%) than alemtuzumab-treated patients in the UKALL14 sub-study. This discrepancy may partly be explained by the higher proportion of MUD transplants in our study, suggesting a role of additional factors not controlled for by the propensity matching criteria employed. However, there were also other significant differences in the GVHD prophylaxis between the two serotherapy groups, with the ATG group also receiving a higher rate of additional agents such as methotrexate and MMF compared with the alemtuzumab group, who predominantly received single-agent cyclosporine.

To date, ATG and alemtuzumab have not been compared in a randomized manner in patients with ALL or other malignant diseases. Two retrospective comparisons of these two types of TCD in the context of RIC and unrelated donor HCT were restricted to patients with myeloid malignancies [25, 26]. Our finding of a nearly identical CIR with ATG and alemtuzumab differs from the results of these retrospective studies conducted primarily in AML and MDS patients, which showed a higher relapse rate with alemtuzumab than with ATG. A major difference between these studies is the inclusion of patients with less than a 10/10 HLA match and beyond CR1 in the study reported by Robin et al. [26], which also used a higher alemtuzumab dose (100 mg in 80% of patients), compared with 50 or 60 mg in 85% of patients in our study. Robin et al. also reported significantly lower rates of acute and chronic GVHD with alemtuzumab than with ATG, possibly due to use of a higher dose of alemtuzumab. Similar to the latter study, alemtuzumab was associated with a significantly higher CIR and corresponding lower CI of grade II-IV acute GVHD, but not of chronic GVHD than ATG in the study reported by Forcade et al. [25]. No information on doses of TCD regimens was provided, and most patients in this study had MDS, 40% of whom were not in CR. Moreover, only 21–34% of patients received a MUD graft, in contrast to our study, which included MUD HCT exclusively. The substantial differences between these studies seem sufficient to explain the discordant results regarding impact of ATG and alemtuzumab on relapse rate.

Notably, neither we nor the two studies comparing ATG and alemtuzumab in patients with myeloid malignancies found significant differences in NRM. The inferior GRFS and OS with alemtuzumab reported by Forcade et al. was caused by the higher relapse incidence, which counterbalanced the lower rate of acute GVHD [25]. This effect was observed neither in our study nor that of Robin et al., both of which used propensity matching and did not detect an impact of TCD regimen on OS or GRFS. The superior RFS with ATG versus alemtuzumab shown by Robin et al. was most pronounced in CMV-positive patients [26], suggesting a negative, but dose-dependent effect of alemtuzumab on viral infections [27]. Because of limited patient numbers, we did not conduct a separate analysis by CMV status, and multivariable analysis was precluded by propensity score matching for methodological reasons.

Optimal doses of ATG and alemtuzumab should efficiently control GVHD without increasing the risk of viral infections and leukemic relapse and may vary according to stem cell source, conditioning regimen and GVHD prophylaxis [28]. In a retrospective study of MUD HCT using FMA conditioning, alemtuzumab doses of 60 and 100 mg, proved equally effective in preventing severe GVHD [29]. An even lower dose of 40 mg will be administered to patients transplanted from a MUD within the ongoing randomized ALL-RIC trial comparing fludarabine and melphalan with a TBI 8 Gy and cyclophosphamide [30]. Regarding ATG, doses of 5–10 mg thymoglobulin and 30 mg/kg Grafalon mostly been used in the setting of RIC HCT (summarized in [28]), but randomized trials are not available.

Future treatment decisions will also need to consider post-transplant cyclophosphamide (PT-CY) as an option for in vivo TCD based on a recent retrospective analyses comparing ATG with PT-CY in ALL patients in a similar clinical setting as in our study [31]. Compared to our patient population, patients in the study by Giebel et al. were younger (40 vs 56 years) and mostly treated with myeloablative conditioning. In multivariate analysis, PT-CY was associated with significantly improved 2-year LFS (71.0% vs 59.0%, HR (95% CI) 1.57 (1.01–2.45)) and reduced extensive chronic GVHD (10% vs 17%, HR 0.54 (0.3–0.98) combined with a lower relapse incidence (18% vs 25%, *p* = 0.045) in univariate analysis. The results of an ongoing prospective randomized trial comparing these two IS modalities are eagerly awaited, but will not provide additional information on the comparative role of alemtuzumab-based transplant strategies in ALL.

Limitations of our study include its retrospective design, differences in definitions of what constitutes a high-risk ALL as indication for an allogeneic transplant in the contributing countries, and the paucity of information on the use of donor lymphocyte infusions and of MRD data [32]. The latter was due to the fact that MRD was not captured in the EMBT database until more recently. While the proportion of patients in which MRD data was unavailable and the subset composition of ALL was exactly matched between the ATG and alemtuzumab cohorts, we cannot rule out that differences in MRD levels may have caused a bias with respect to relapse rate. Differences in dosing of ATG and alemtuzumab as well as use of different ATG preparations are further limitations of our analysis. In view of the geographically disparate usage of ATG and alemtuzumab, it is almost certain that no prospective randomized trial of sufficient size comparing these two strategies will ever be conducted. Moreover, international multicenter studies still have the disadvantage that MRD monitoring is often not performed in a standardized manner[33]. Lastly, the registry data do not include information on use of TKI maintenance in the subset of Philadelphia positive ALL patients.

Our study demonstrates that alemtuzumab and ATG-based prophylaxis of GVHD result in comparable survival outcomes, CIR, NRM and incidence of acute and chronic GVHD in patients with high-risk ALL in CR1 who underwent HCT from matched unrelated donors. While GVHD and infections continue to be the main reasons for treatment failure and death in both groups, we conclude that both IS strategies are valid approaches in the context of various RIC regimens for this population of ALL patients.

## Supplementary information

Supplementary Table 1

Supplementary Information

## Data Availability

GB, ML, FC, and MM had full access to all study data (available upon data-specific request).
