# Supplementary material for: Anti-thymocyte globulin (ATG)- or alemtuzumab-based graft-versus-host disease prophylaxis in reduced-intensity conditioning allogeneic hematopoietic cell transplantation (HCT) for patients 40 years and older with acute lymphoblastic leukemia in first complete remission: a study from the EBMT Acute Leukemia Working Party
Source: Bone Marrow Transplant. 2026 Mar 6;61(4):462–8. doi: 10.1038/s41409-026-02805-4 (PMC13056518; doi:10.1038/s41409-026-02805-4)
Supplement: Supplementary file 1 — Supplementary Table 1 [file 41409_2026_2805_MOESM1_ESM.docx]

**Supplementary Table 1**: Demographics and transplant modalities for all patients (n=357) according to *in vivo* T cell depletion, showing imbalance between groups

|  |  | **ATG**  **(n=236)** | **Alemtuzumab (n=121)** | **P** |
| --- | --- | --- | --- | --- |
|  |  |  |  |  |
| Median follow-up (months) | median [IQR] | 36.38  [32.64-41.15] | 50.65  [47.92-59.79] | 0.03 |
|  |  |  |  |  |
| Patient age (years) | median (min-max) [IQR] | 60.4 (40.6-72.2) [55.4-63.8] | 53.6 (40.9-71) [46.9-58.4] | <0.0001 |
|  |  |  |  |  |
| Diagnosis | Ph neg B ALL | 56 (23.7%) | 57 (47.1%) | <0.0001 |
|  | Ph pos B ALL | 143 (60.6%) | 42 (34.7%) |  |
|  | T ALL | 37 (15.7%) | 22 (18.2%) |  |
|  |  |  |  |  |
| Year transplant | median (min-max) | 2016  (2010-2021) | 2016  (2010-2021) | 0.85 |
|  |  |  |  |  |
| Time diagnosis to HCT (mo) | median (min-max) [IQR] | 6.3 (2.7-21.7) [5.2-7.8] | 6 (3-21.4)  [4.9-7.3] | 0.29 |
|  | missing | 2 | 0 |  |
|  |  |  |  |  |
| Patient sex | Male | 116 (49.2%) | 61 (50.4%) | 0.82 |
|  | Female | 120 (50.8%) | 60 (49.6%) |  |
|  |  |  |  |  |
| Donor sex | Male | 171 (74.7%) | 85 (72%) | 0.6 |
|  | Female | 58 (25.3%) | 33 (28%) |  |
|  | missing | 7 | 3 |  |
|  |  |  |  |  |
| Female to male combination | no F->M | 212 (91.4%) | 101 (84.2%) | 0.041 |
|  | F->M | 20 (8.6%) | 19 (15.8%) |  |
|  | missing | 4 | 1 |  |
|  |  |  |  |  |
| Patient CMV | negative | 67 (28.5%) | 62 (52.5%) | <0.0001 |
|  | positive | 168 (71.5%) | 56 (47.5%) |  |
|  | missing | 1 | 3 |  |
|  |  |  |  |  |
| Donor CMV | negative | 130 (56.5%) | 70 (58.8%) | 0.68 |
|  | positive | 100 (43.5%) | 49 (41.2%) |  |
|  | missing | 6 | 2 |  |
|  |  |  |  |  |
| Karnofsky score | <90 | 60 (26.9%) | 44 (38.6%) | 0.028 |
|  | >=90 | 163 (73.1%) | 70 (61.4%) |  |
|  | missing | 13 | 7 |  |
|  |  |  |  |  |
| HCT-CI | HCT-CI = 0 | 85 (45.9%) | 65 (54.2%) | 0.21 |
|  | HCT-CI = 1 or 2 | 47 (25.4%) | 31 (25.8%) |  |
|  | HCT-CI >=3 | 53 (28.6%) | 24 (20%) |  |
|  | missing | 51 | 1 |  |
|  |  |  |  |  |
| MRD pre HSCT | MRD negative | 101 (63.5%) | 32 (71.1%) | 0.35 |
|  | MRD positive | 58 (36.5%) | 13 (28.9%) |  |
|  | missing | 77 | 76 |  |
|  |  |  |  |  |
| Details conditioning | BuCy | 2 (0.8%) | 0 (0%) |  |
|  | BuFlu | 113 (47.9%) | 0 (0%) |  |
|  | TBF | 28 (11.9%) | 0 (0%) |  |
|  | FluMel | 27 (11.4%) | 115 (95%) |  |
|  | FTM | 1 (0.4%) | 0 (0%) |  |
|  | FluTreo | 5 (2.1%) | 0 (0%) |  |
|  | Flucy | 4 (1.7%) | 0 (0%) |  |
|  | Cy-TBI | 0 (0%) | 1 (0.8%) |  |
|  | Flu-TBI | 42 (17.8%) | 0 (0%) |  |
|  | Bu-TBI | 1 (0.4%) | 0 (0%) |  |
|  | FLAMSA-TBI | 1 (0.4%) | 0 (0%) |  |
|  | Thiotepa-based | 2 (0.8%) | 2 (1.7%) |  |
|  | Clofarabin-based | 7 (3%) | 0 (0%) |  |
|  | Other chemotherapy | 3 (1.3%) | 3 (2.5%) |  |
|  |  |  |  |  |
| GVHD prevention | CSA | 27 (11.4%) | 91 (75.2%) |  |
|  | MTX | 1 (0.4%) | 0 (0%) |  |
|  | Tacrolimus | 2 (0.8%) | 7 (5.8%) |  |
|  | MMF | 1 (0.4%) | 1 (0.8%) |  |
|  | CSA+MTX | 86 (36.4%) | 16 (13.2%) |  |
|  | Tacrolimus+MTX | 7 (3%) | 0 (0%) |  |
|  | CSA+MMF | 87 (36.9%) | 6 (5%) |  |
|  | CSA+MTX+MMF | 5 (2.1%) | 0 (0%) |  |
|  | Tacrolimus+MMF | 15 (6.4%) | 0 (0%) |  |
|  | Sirolimus+MMF | 1 (0.4%) | 0 (0%) |  |
|  | CSA+MMF+  Tacrolimus | 1 (0.4%) | 0 (0%) |  |
|  | Tacrolimus+  Sirolimus | 3 (1.3%) | 0 (0%) |  |

**Abbreviations:** ATG, anti-thymocyte globulin; HCT, hematopoietic cell transplantation; ALL, acute lymphoblastic leukemia; Ph; Philadelphia; MRD, measurable residual disease; CSA, cyclosporine A; MTX, methotrexate; MMF, mycophenolate mofetil; Bu; busulfan; Cy, cyclophosphamide; Flu, fludarabine; TBF, thiotepa, busulfan, fludarabine; Mel, melphalan; Treo, treosulfan; TBI, total body irradiation; FLAMSA, fludarabine, amsacrine, cytarabine
