# Supplementary material for: Anti-thymocyte globulin (ATG)- or alemtuzumab-based graft-versus-host disease prophylaxis in reduced-intensity conditioning allogeneic hematopoietic cell transplantation (HCT) for patients 40 years and older with acute lymphoblastic leukemia in first complete remission: a study from the EBMT Acute Leukemia Working Party
Source: Bone Marrow Transplant. 2026 Mar 6;61(4):462–8. doi: 10.1038/s41409-026-02805-4 (PMC13056518; doi:10.1038/s41409-026-02805-4)
Supplement: Supplementary file 2 — Supplementary Information [file 41409_2026_2805_MOESM2_ESM.docx]

Supplementary Information: Contributing institutions

Nottingham City Hospital, Nottingham, United Kingdom; Karolinska University Hospital, Stockholm, Sweden; Southampton General Hospital, Southampton, United Kingdom; Birmingham Centre for Cellular Therapy and Transplant (BCCTT), Birmingham, United Kingdom; Kings College Hospital London, London, United Kingdom; Programme de Transplantation & Therapie Cellulaire, Marseille, France; University Hospitals Bristol and Weston NHSFT, Bristol, United Kingdom; Sheffield Royal Hallamshire, Sheffield, United Kingdom; CHU de Lille, Lille, France; Glasgow Royal Infirmary, Glasgow, United Kingdom; Addenbrookes Hospital Cambridge, Cambridge, United Kingdom; University Hospital Gasthuisberg, Leuven, Belgium; Royal Marsden Hospital, London, United Kingdom; University College London Hospital, London, United Kingdom; Hope Directorate - St. James's Hospital, Dublin, Ireland; University Hospital Uppsala, Uppsala, Sweden; CHU Grenoble Alpes - Universite Grenoble Alpes, Grenoble, France; Leicester Royal Infirmary, Leicester, United Kingdom; Tor Vergata University of Rome, Rome, Italy; Saint-Louis Hospital, BMT Unit, Paris, France; IRCCS Azienda Ospedaliero-Universitaria di Bologna, Bologna, Italy; CHU Nantes, Nantes, France; CHU Bordeaux, Hopital Haut-Leveque, Pessac, France; Sahlgrenska University Hospital, Goeteborg, Sweden; A.Z. Sint-Jan, Brugge, Belgium; Olomouc University Hospital, Olomouc, Czech Republic; ASST Papa Giovanni XXIII, Bergamo, Italy; CHU Lapeyronie, Montpellier, France; CHRU Limoges, Limoges, France; University Hospital Basel, Basel, Switzerland; Medizinische Universitaet Wien, Vienna, Austria; Cliniques Universitaires St. Luc, Brussels, Belgium; University Medical Centre Utrecht, Utrecht, Netherlands; CHU CAEN, Caen, France;Skanes University Hospital, Lund, Sweden; Cardiff University Hospital of Wales & Swansea, Cardiff, United Kingdom; Azienda Ospedaliera Universitaria Careggi, Firenze, Italy; Singapore General Hospital, Singapore, Singapore; Clatterbridge Cancer Centre Liverpool, Liverpool, United Kingdom; Del-pesti Centrumkorhaz, Budapest, Hungary; Hospital Universitario Donostia, S Sebastian, Spain; Gustave Roussy Cancer Campus, Villejuif, France; CHRU NANCY, Vandoeuvre les Nancy, France; Charles University Hospital, Hradec, Czech Republic; Umea University Hospital, Umea, Sweden; Ghent University Hospital, Gent, Belgium; Hopital Saint Antoine, Paris, France; Medizinische Klinik m. S. Hamatologie, Onkologie und Tumorimmunologie, Berlin, Germany; University of Freiburg, Freiburg, Germany; Centre Henri Becquerel, Rouen, France; Antwerp University Hospital (UZA), Antwerp, Belgium; Klinikum Augsburg, Augsburg, Germany; University Hospital, Zurich, Switzerland; Hospital Sirio-Libanes, Sao Paulo, Brazil; Oslo University Hospital, Rikshospitalet, Oslo, Norway; Hospital U. Marques de Valdecilla, Santander, Spain; Institut de Cancerologie Lucien Neuwirth, Saint Etienne, France; St James University Hospital Leeds, Leeds, United Kingdom; University Medical Center Schleswig-Holstein, Campus Kiel, Kiel, Germany; Hopital La Miletrie, Poitiers, France; CHU ESTAING, Clermont, France; RVI Newcastle, Newcastle, United Kingdom; Inst. Portugues de Oncologia do Porto, Porto, Portugal; University Hospital Center Rebro, Zagreb, Croatia; Universitaetsklinikum Magdeburg, Magdeburg, Germany; HUCH Comprehensive Cancer Center, Helsinki, Finland; Department of Hematology and Transplantology of Lower Silesian Center of Oncology, Wroclaw, Poland; Elisabethinen Hospital, Linz, Austria; Manchester Royal Infirmary, Manchester; United Kingdom; Institute of Hematology and Blood Transfusion, Prague, Czechia; Centre Hospitalier Universitaire de Rennes, Rennes, France; Universitaetsklinikum Wuerzburg, Wuerzburg, Germany; Charles University Hospital, Plzen, Czech Republic; University Hospital Linkoeping, Linkoeping, Sweden; Christie Hospital Manchester, Manchester, United Kingdom; Derriford Hospital Plymouth, Plymouth, United Kingdom; National Research Center for Hematology, Moscow, Russian Federation; ZSIS Universitaetsklinikum Knappschaftskrankenhaus Bochum GmbH, Bochum, Germany; Universitaet Bonn, Bonn, Germany; Hopital Necker Adults, Paris, France; Hospital Guglielmo da Saliceto, Piacenza, Italy; Institut Jules Bordet, Brussels, Belgium; Universitaet Tuebingen, Tuebingen, Germany; Univ. La Sapienza, Rome, Italy; Hopital Jean Minjoz, Besancon, France; Nijmegen Medical Centre, Nijmegen, Netherlands; Universite Paris IV, Hopital la Pitie-Salpetriere, Paris, France; Research Committee - University of Patras, Patras, Greece; Klinikum Karlsruhe gGmbH, Karlsruhe, Germany; Hospital San Maurizio, Bolzano, Italy; Ospedale Infantile Regina Margherita, Torino, Italy; U.O.S.A Centro Trapianti e Terapia Cellulare, Siena, Italy; ZNA, Antwerp, Belgium; Rambam Medical Center, Haifa, Israel; University Hospital Aachen, Aachen, Germany; Istituto Clinico Humanitas, Milano, Italy; Medical Clinic and Policinic 1, Leipzig, Germany; Istanbul Medipol University, Istanbul, Turkey; Ospedale Dell'Angelo, Venezia, Italy; HELIOS Klinikum Duisburg, Duisburg, Germany; CHU Nice - Hopital de l'ARCHET I, Nice, France; University of Heidelberg, Heidelberg, Germany; University of Cologne, Cologne, Germany; St. George`s Hospital London, London, United Kingdom; Ospedale San Gerardo, Monza, Italy; TUM Universitatsklinikum, Klinikum rechts der Isar, Munich, Germany; University Hospital Maastricht, MAASTRICHT, Netherlands; University Hospital Brno, Brno, Czech Republic; Az. Ospedaliera S. Croce e Carle, Cuneo, Italy; CHU - Institut Universitaire du Cancer Toulouse, Toulouse, France; Universitair Ziekenhuis Brussel, Brussels, Belgium; CHRU, Angers, France; Hospital C. Panico, Tricase, Italy; C.H.R.U de Brest, Brest, France; Silesian Medical Academy, Katowice, Poland; Ospedale La Maddalena - Dpt. Oncologico, Palermo, Italy; Institute of Hematology and Transfusion Medicine, Warsaw, Poland; RM Gorbacheva Research Institute, Pavlov University, St. Petersburg, Russian Federation; University of Liege, Liege, Belgium; Institut Catala d'Oncologia, Hospital Duran i Reynals, Barcelona, Spain; University Medical Center Mainz, Mainz, Germany; IRCCS San Raffaele Hospital Scientific Institute, Milano, Italy; Hospital Gregorio Maranon, Madrid, Spain; St. Franziskus Hospital, Flensburg, Germany.
